# Prognostic markers in pheochromocytomas/paragangliomas: the role of histopathology, SDHB, MAML3 and MCM6 expression

**DOI:** 10.1007/s40618-025-02610-8

**Published:** 2025-05-30

**Authors:** Emel Yaldır, Balça Begüm Cengiz, Osman Furkan Mülkem, Muzaffer Bilgin, Mustafa Fuat Açıkalın

**Affiliations:** 1 Faculty of Medicine, Department of Pathology, Eskişehir Osmangazi University, Eskişehir, Turkey; 2 Department of Pathology, Kütahya Health Sciences University Evliya Çelebi Education and Research Hospital, Kütahya, Turkey; 3 Faculty of Medicine, Department of Pathology, Eskişehir Osmangazi University, Eskişehir, Turkey; 4 Faculty of Medicine, Department of Biostatistics, Eskisehir Osmangazi University, Eskişehir, Turkey; 5 Faculty of Medicine, Department of Pathology, Eskişehir Osmangazi University, Eskişehir, Turkey

**Keywords:** Paraganglioma, Pheochromocytoma, MCM6, SDHB, MAML3, Metastasis

## Abstract

**Objective:**

There are currently no definitive prognostic markers that accurately predict malignant behavior in pheochromocytoma/paraganglioma (PCC/PGL). While metastasis develops in only 10–15% of adrenal-origin, this rate can rise up to 50% in those with extra-adrenal localization. This study aims to investigate the potential predictive effect of known histopathological features in PCC/PGL, alongside SDHB, S100, Ki-67 proliferation index, and the expression of MAML3 and MCM6 in predicting metastatic disease.

**Methods:**

The specimens of 71 patients who were diagnosed with PCC/PGL and underwent total excision between 2010 and 2021 were re-examined. Demographic, clinical, and histopathological data, as well as immunohistochemical results for Ki-67, S100, SDHB, MCM6, and MAML3, were recorded.

**Results:**

While distant organ metastasis was observed in 3.4% (*n* = 1/29) of PCC and 21.2% (*n* = 7/33) of head and neck PGL, this rate was found to be significantly higher at 66.7% (*n* = 6/9) in abdominal PGL (*p* < 0.001). No MAML3 overexpression was observed in any of the cases. Distant organ metastasis was more frequently detected in cases with MCM6 overexpression.

**Conclusions:**

Although there is still no definitive feature that predicts metastasis, in line with the literature, extra-adrenal localization, vascular invasion, capsular invasion, nuclear pleomorphism, hyperchromasia, and confluent necrosis were found to be associated with distant organ metastasis in our study. Additionally, in the multivariate analysis, larger tumor size (> 5.1 cm), the presence of > 3/10 HPF mitosis, and SDHB loss were associated with lower metastasis-free survival. While no conclusions could be drawn regarding MAML3, the prognostic value of MCM6 appears promising.

## Introduction

Pheochromocytoma (PCC) and paraganglioma (PGL) are rare neuroendocrine tumors that arise from the adrenal medulla and extra-adrenal ganglia, respectively. Most are associated with catecholamine hypersecretion, which can lead to significant morbidity and mortality [1]. Today, the majority of PCC/PGLs are diagnosed incidentally during unrelated examinations, followed by cases presenting with symptoms of catecholamine excess, and thirdly, during screenings of patients with known familial syndromes (such as MEN2, von Hippel-Lindau syndrome, neurofibromatosis type 1, or mutations in genes like succinate dehydrogenase [SDH], TMEM-127, MAX, FH, EPAS1/HIF-2α, or MDH2) [2]. Elevated epinephrine levels are observed exclusively in PCC, whereas both epinephrine and norepinephrine increases can be seen in PGL [3]. Currently, there are no definitive prognostic markers to accurately predict the malignant behavior of primary tumors. The 2017 World Health Organization (WHO) classification recommends replacing the terms “benign” and “malignant” with “non-metastatic” and “metastatic” terminology. Metastasis is defined by the presence of tumors in regions devoid of normal chromaffin cells [4]. While only 10–15% of PCCs metastasize, this rate rises to 50% in PGLs [5].

Due to the lack of reliable data predicting metastasis, PGL/PCC patients require lifelong follow-up, as metastasis can occur even after 20 years or more [1]. Some studies have identified factors associated with a higher risk of metastasis, including larger tumor size, extra-adrenal origin, and methoxytyramine secretion [6, 7]. The presence of a germline pathogenic variant in SDHB is a well-established risk factor for metastasis, although approximately half of patients with metastatic disease do not have a hereditary SDHB pathogenic variant [8]. Other studies have found mutations associated with metastatic disease, such as ATRX, a pseudohypoxia mRNA expression subtype, TERT alteration, and MAML3 fusion anomalies [9–11]. In cases of PCC/PGL with MAML3 fusion, nuclear overexpression of the protein has been detected immunohistochemically, supporting a metastatic potential [12].

Several scoring systems have been developed to assess metastatic potential in these tumors. Systems like Pheochromocytoma of the Adrenal gland Scaled Score (PASS) [13], Grading of Adrenal Pheochromocytoma and Paraganglioma (GAPP) system [14], and COPPS [15] combine various clinical and histopathological features to predict the clinical course of PCC/PGL. However, no scoring system has yet been recommended by the WHO [4].

Recently, the minichromosome maintenance (MCM) protein complex, consisting of six highly conserved proteins (MCM2-7), has been highlighted for its prognostic significance in several cancers. This complex plays a crucial role in the initiation of DNA replication. MCM6 expression has been studied in various human cancers, including clear cell renal cell carcinoma [16]. These studies reported MCM6 overexpression in cancer tissues and its association with key clinicopathological factors [16]. To our knowledge, there is only one study investigating the prognostic significance of MCM6 protein expression in PCC/PGL [15].

This study aims to explore the potential predictive effects of MAML3 and MCM6 expression, in addition to known histopathological features, SDHB, S100, and the Ki-67 proliferation index, in forecasting metastatic disease in PCC and PGL.

## Material and method

A total of 71 patients diagnosed with PCC and PGL between 2010 and 2021, who underwent total excision, were included in this study. The study was approved by the Non-invasive Clinical Research Ethics Committee of Eskişehir Osmangazi University (E-25403353-050.99-2300070893).

### Pathological evaluation

All cases were re-evaluated blindly by two pathologists. Histopathological characteristics such as tumor location, tumor size, presence of large cell nests, pseudorosette formation, cellularity, monotony, cellular spindling, mitotic count, presence of atypical mitoses, adipose tissue extension, capsular invasion, vascular invasion, hyperchromasia, pleomorphism, and presence of confluent necrosis were categorized and recorded. The previously assessed Ki-67 proliferation index was re-examined using immunohistochemical slides. Archived tumor blocks were retrieved, and immunohistochemical markers for S100, SDHB, MCM6, and MAML3 were applied.

The Ki-67 proliferation index was calculated as a percentage by counting at least 500 cells in hot spots under the highest magnification. Cases were categorized into three groups based on their Ki-67 index: <1%, 1–3%, and > 3%, according to GAPP [14].

Loss of S100 was defined as absent or diffuse reduction in sustentacular cells. The presence or absence of S100 loss was recorded. Loss of SDHB expression was defined by the absence of cytoplasmic staining in all tumor cells, and results were recorded as either present of loss or absent of loss.

For the MAML3 immunohistochemical marker, nuclear expression was recorded as positive or negative.

MCM6 immunohistochemical staining was calculated by counting at least 500 cells in hot spots under the highest magnification. All nuclear staining, regardless of intensity, was considered positive. The percentage of positive cells was scored from 0 to 4, corresponding to 0%, 1–25%, 26–50%, 51–75%, and 76–100%, respectively. Staining intensity was scored from 0 to 3, as negative, weak, moderate, or strong. Using these two scores, an overall score ranging from 0 to 12 was calculated. Cases with scores between 0 and 3 were classified as negative, and those with scores above 3 were considered positive [17, 18].

### Immunohistochemical analysis

Four-micrometer-thick tissue sections were taken from the selected paraffin-embedded blocks and mounted on slides. The slides were deparaffinized and rehydrated using xylene and ethanol. Endogenous peroxidase was blocked using 0.3% H2O2 in phosphate-buffered saline. Heat-induced antigen retrieval was performed by boiling tris-ethylenediaminetetraacetic acid buffer for 15 min. S100 Protein Ab-1 (4 C4.9), SDHB [EPR10880], Anti-MAML3 antibody [MAML3/1303], and MCM6 Antibody (EP375), diluted in normal antibody diluent, were incubated at room temperature for 2 h. Antibody visualization was performed using the EnVision kit (Dako/Agilent), and slides were counterstained with hematoxylin. Positive external controls were used for each immunohistochemical marker.

### Clinical evaluation

Patient age, gender, catecholamine levels, presence of distant organ metastasis, site of metastasis, bilaterality of primary, clinical follow-up (months), time to metastasis (months), and death status were recorded. Genetic counseling information was unavailable for the cases. Catecholamine levels were not available for most cases other than those with PCC.

### Statistical analysis

Continuous data are presented as Mean ± Standard Deviation, while categorical data are presented as percentages (%). The Shapiro-Wilk test was used to assess the normality of the data distribution. The Mann-Whitney U test was used for comparing groups that did not follow a normal distribution. Pearson Chi-Square, Pearson Exact Chi-Square, and Fisher’s Exact Chi-Square tests were used to analyze cross-tabulated data. Cox regression analysis was performed to identify risk factors. Receiver Operating Characteristic (ROC) analysis was used to determine appropriate cut-off points for independent markers and to calculate sensitivity and specificity values. Analyses were conducted using the R Core Team (2024). *R: A Language and Environment for Statistical Computing*. R Foundation for Statistical Computing, Vienna, Austria. https://www.R-project.org/. A p-value of < 0.05 was considered statistically significant.

## Results

### Patient characteristics

The study included a total of 71 patients with a mean age of 53.15 years (range: 12–74), 67.6% (*n* = 48) of whom were female. Tumors originated from the head and neck region in 46.5% (*n* = 33), the adrenal glands in 40.8% (*n* = 29), and the abdomen in 12.7% (*n* = 9). In most cases of PCC, catecholamine levels were elevated, whereas this was not the case in most PGL cases. In 86.2% of PCC cases (n: 25), elevated catecholamine levels were observed, with epinephrine elevated in 40% of cases, norepinephrine in 72%, and dopamine in 36%. In contrast, only 21.4% of PGL cases (n: 9) showed elevated catecholamine levels, with no cases exhibiting elevated epinephrine, 22.2% showing elevated dopamine, and 33.3% showing elevated norepinephrine. In PGL cases, the elevation of epinephrine and norepinephrine was significantly lower compared to PCC cases (0% vs. 40%, *p* = 0.024; 33.3% vs. 72%, *p* = 0.041, respectively). No significant correlation was found between dopamine expression and tumor localization. Distant organ metastasis was detected in 19.7% (*n* = 14) of the patients during follow-up. Metastasis occurred in 3.4% (*n* = 1/29) of PCCs, 21.2% (*n* = 7/33) of head and neck PGLs, and 66.7% (*n* = 6/9) of abdominal PGLs, showing a significantly higher metastasis rate in abdominal PGLs (*p* < 0.001). No significant correlation was found between distant organ metastasis and catecholamine levels. The metastatic sites were as follows: bone (8, 57.1%), liver (4, 28.5%), lung (4, 28.5%), and bladder (1, 7.1%), with lymph node metastasis present in 8 (57.1%) cases. None of the cases exhibited bilateral involvement. The mean follow-up duration was 101.3 months (range: 37–264), and disease-related mortality was recorded in 9.9% (*n* = 7) of the patients. The demographic and clinical data according to the localization of the patients are summarized in Table 1.

**Table 1:** Demographic and clinical data of patients according to tumor localization

	Age Median (Min-Max)	Gender F/M	Distant organ metastasis *n* (%)	Clinical follow-up (m) Median (Min-Max)	Time to metastasis (m) Median (Min-Max)	Death status *n* (%)	Total *n* (%)
PCC	54 (23–71)	14/15	1 (3.4)	96 (39–177)	3	0 (0)	29
Abdominal PGL	54 (12–72)	1/6	6 (66.7)	78 (37–205)	35.50 (0–58)	4 (44.4)	9
Head & Neck PGL	59 (25–74)	31/2	7 (21.2)	94 (40–264)	50 (0–171)	3 (9.1)	33

### Histopathological features

Large cell nests were observed in 46.5% of cases, diffuse growth patterns in 15.5%, pseudorosette formation in 5.6%, high cellularity (> 250 cells/1 high-power field [HPF]) in 31%, cellular monotony in 9.9%, cellular spindling in 14.1%, increased mitoses (> 3/10 HPF) in 11.3%, atypical mitoses in 14.1%, adipose tissue extension in 12.7%, vascular invasion in 45.1%, capsular invasion in 63.4%, marked nuclear pleomorphism in 32.4%, nuclear hyperchromasia in 32.4%, and confluent necrosis in 16.9%. The mean tumor size was recorded as 4.42 cm (range: 1–13). No significant differences were found between PCC and PGL in most histopathological features. Only diffuse growth pattern, cellular spindling, and adipose tissue extension were significantly more frequent in PCC cases (*p* = 0.019, *p* = 0.043, *p* = 0.002, respectively). 

**Fig. 1:**
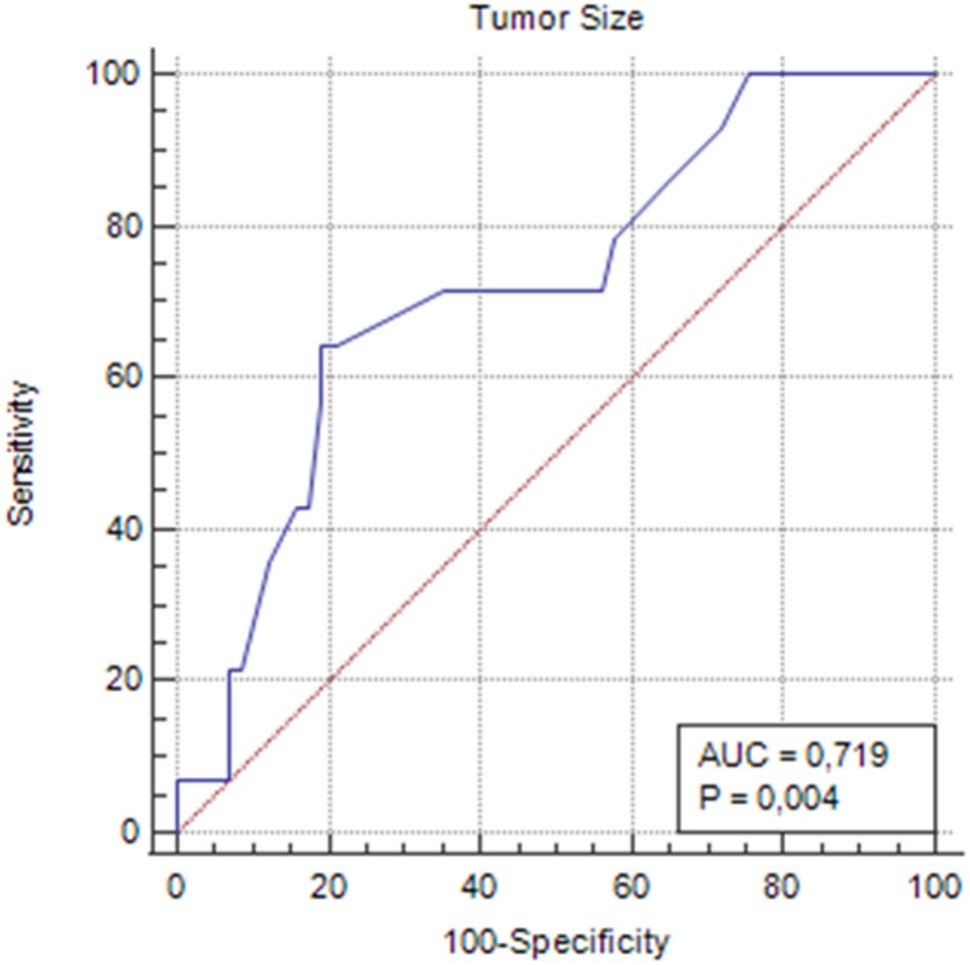
Cut-off analysis of tumor size associated with organ metastasis status, receiver operating characteristic curve. Area under the curve = 0.719; *p* = 0.004. Sensitivity = 64.29%; specificity = 80.70%; and cut-off > 5.1 cm

Statistical analysis revealed a significant association between distant organ metastasis and the following factors: mitoses > 3/10 HPF (*p* = 0.006), vascular invasion (*p* = 0.012), capsular invasion (*p* = 0.025), nuclear pleomorphism (*p* = 0.027), nuclear hyperchromasia (*p* = 0.009), and the presence of confluent necrosis (*p* = 0.036). Other histopathological features did not show a statistically significant relationship with metastasis. Tumor size was also significantly associated with metastasis, with a mean tumor diameter of 5.73 cm in metastatic cases compared to 4.10 cm in non-metastatic cases (*p* = 0.011). The relationship between histopathological features and distant organ metastasis is summarized in Table 2. A cut-off analysis for tumor size revealed that tumors larger than 5.1 cm were associated with a higher specificity for predicting metastasis (*p* = 0.004) (Fig. 1).

**Table 2:** Histopathological features associated with distant organ metastasis in PCC/PGLs

Metastasis	Absent (*n* = 57)	Present (*n* = 14)	*p* value	Total (*n* = 71)
*Localızatıon n (%)*				
PCC	28 (96.6)	1 (3.4)	**< 0.001**	29 (40.8)
Head & neck PGL	26 (78.8)	7 (21.2)		33 (46.4)
Abdominal PGL	3 (33.3)	6 (66.7)		9 (12.6)
*Mıtosıs n (%)*				
≤ 2/10 HPF	54 (85.7)	9 (14.3)	**0.006**	63 (88.7)
> 3/10 HPF	3 (37.5)	5 (62.5)		8 (11.3)
*Vascular ınvasıon n (%)*				
Absent	36 (92.3)	3 (7.7)	**0.012**	39 (54.9)
Present	21 (65.6)	11 (34.4)	**0.012**	32 (45.1)
*Capsular ınvasıon n (%)*				
Absent	25 (96.2)	1 (3.8)	**0.025**	26 (36.6)
Present	32 (71.1)	13 (28.9)		45 (63.4)
*Pleomorphısm n (%)*				
Absent	42 (87.5)	6 (15.5)	**0.027**	48 (67.6)
Present	15 (65.2)	8 (34.8)		23 (32.4)
*Hyperchromasıa n (%)*				
Absent	43 (89.6)	5 (10.4)	**0.009**	48 (67.6)
Present	14 (60.9)	9 (39.1)		23 (32.4)
*Confluent necrosıs n (%)*				
Absent	50 (84.7)	9 (15.3)	**0.036**	59 (83.1)
Present	7 (58.3)	5 (41.7)		12 (16.9)
Tumor sıze, mean (cm) 95% Confidence Interval	4.10 (3.54–4.66)	5.73 (4.21–7.24)	**0.011**	

Statistically significant values are in bold

### Immunohistochemical features

The Ki-67 proliferation index was < 1% in 38% of cases, 1–3% in 26.8%, and > 3% in 35.2%. There was no statistically significant correlation between the Ki-67 index and metastasis or PCC/PGL.

S100 loss was observed in 46.5% of cases, but no significant relationship was found between S100 loss and metastasis. However, PGLs exhibited more frequent S100 loss compared to PCCs (*p* = 0.004), suggesting that S100 loss may be more related to the tumor’s tissue origin than its prognostic significance.

**Fig. 2:**
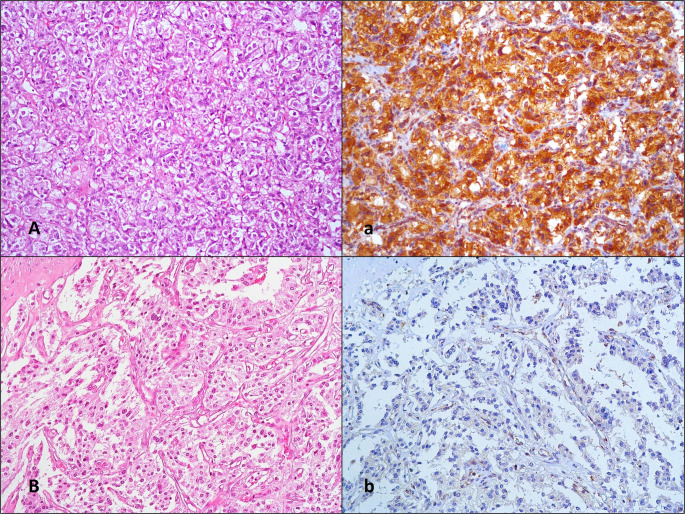
Examples of cases with no loss of SDHB expression (**A, a**) and cases with loss of expression (**B, b**), H&E x200 (**A, B**), immunohistochemical staining x200 (**a, b**)

SDHB expression loss was detected in 21.1% of cases, with a significantly higher incidence of distant organ metastasis in SDHB-negative cases (*p* = 0.037) (Fig. 2). There was no significant difference in SDHB expression loss between PCCs and PGLs.

**Fig. 3:**
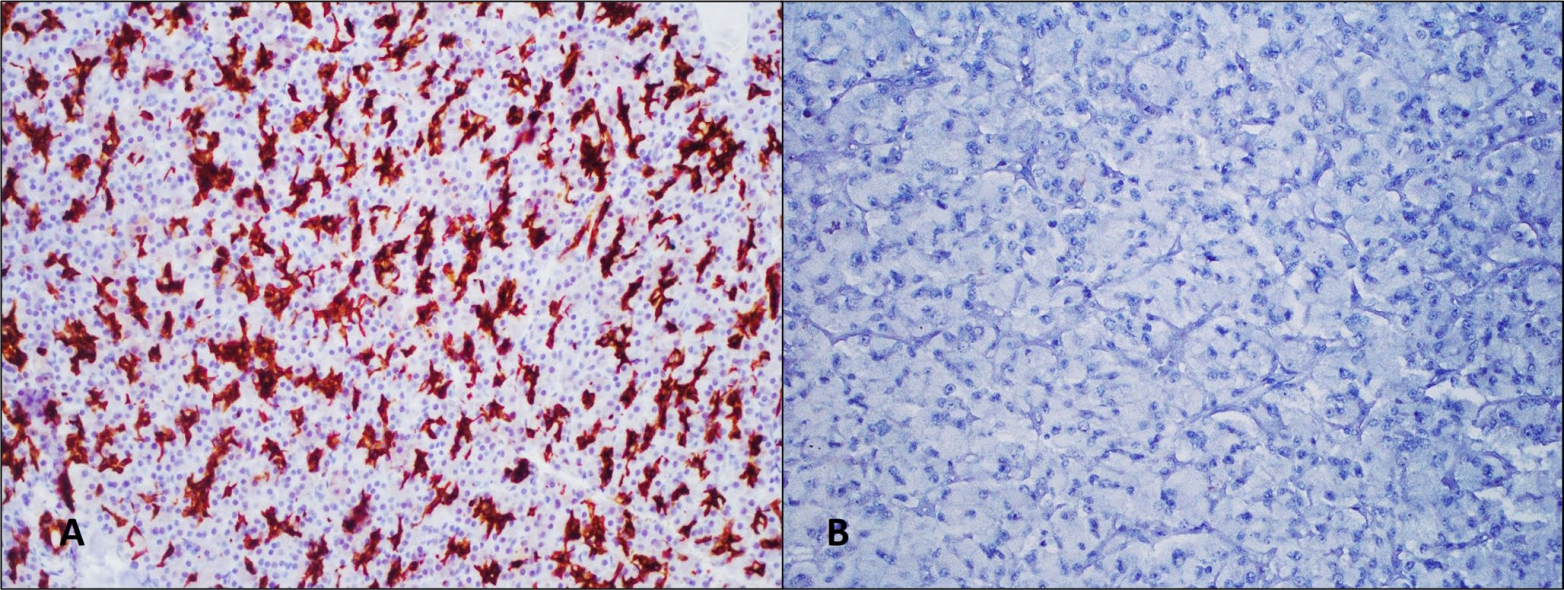
MAML3 negativity (**B**), external positive control (**A**), x200, immunohistochemical staining

No MAML3 overexpression was detected in any of the cases (Fig. 3). Although this suggests that there were no tumors with MAML3 fusion in our series, the absence of molecular confirmation prevents a definitive conclusion on this matter.

**Fig. 4:**
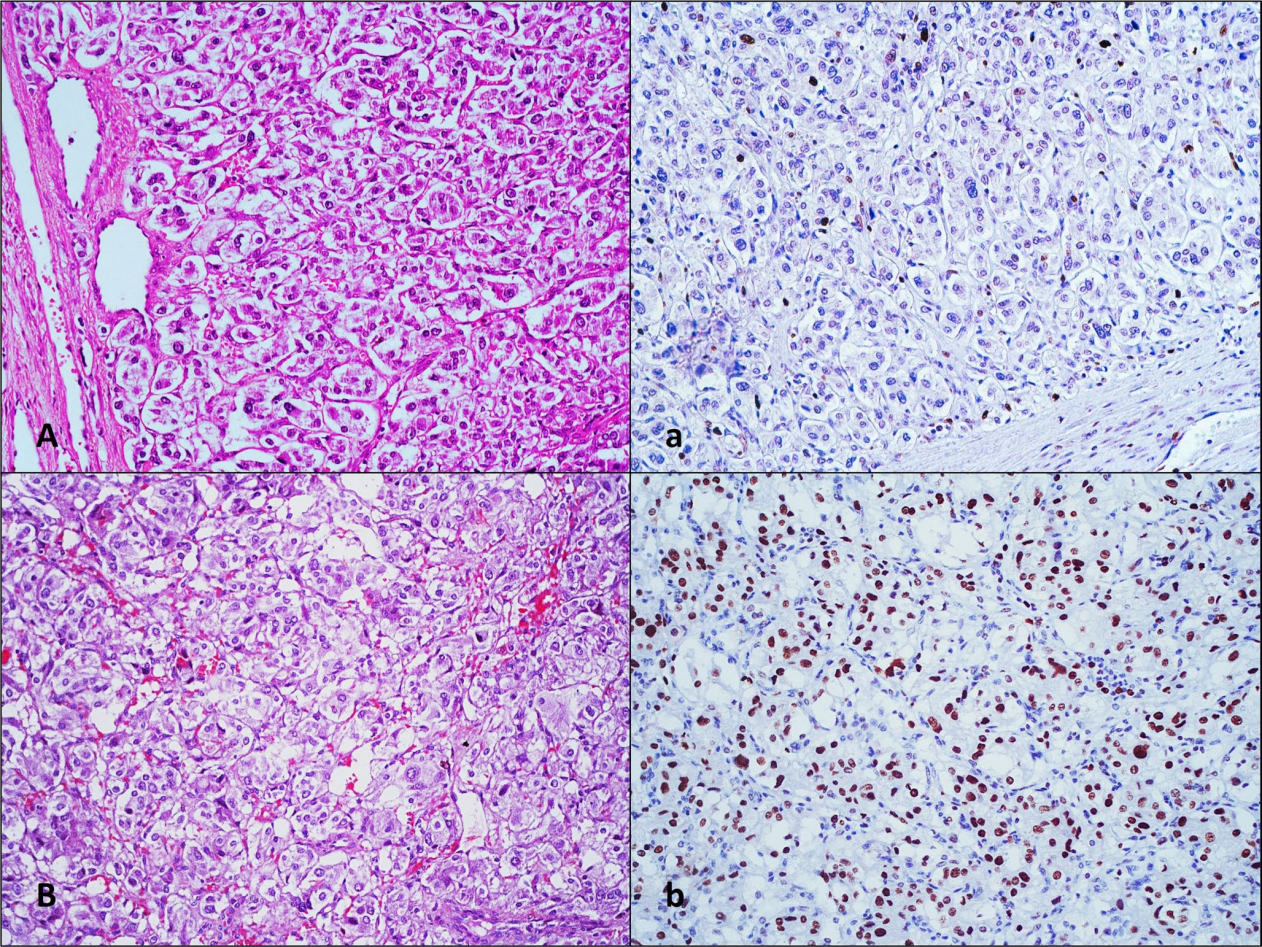
Examples of cases with low expression (overall score ≤ 3) (**A, a**) and high expression (overall score > 3) (**B, b**) detected in MCM6 staining, H&E x200 (**A, B**), immunohistochemical staining x200 (**a, b**)

MCM6 nuclear expression was detected in varying intensities in all tumors. The mean MCM6 expression percentage was 64.3% (range: 13–97), with 16.9% of cases classified as weak (score 1), 28.2% as moderate (score 2), and 54.9% as strong (score 3). Based on percentage of positive cells, 11.3% of cases were scored as 1, 26.8% as 2, 16.9% as 3, and 45.1% as 4. When the overall score, combining percentage and intensity, was calculated, 67.6% of cases (overall score > 3) were classified as positive (Fig. 4). Although cases with positive MCM6 overall scores had a higher incidence of metastasis compared to negative cases (25% vs. 8.7%, respectively), this difference was not statistically significant (*p* = 0.094).

The relationship between immunohistochemical features and distant organ metastasis is summarized in Table 3.

**Table 3:** Relationship between immunohistochemical features and metastasis in PCC/PGLs

Metastasis	Absent (*n* = 57)	Present (*n* = 14)	*P* value	Total (*n* = 71)
*Ki−67 ındex n (%)*				
< 1% 1–3% > 3%	22 (81.5) 17 (89.5) 18 (72.0)	5 (18.5) 2 (10.5) 7 (28.0)	0.346	27 (38.0) 19 (26.8) 25 (35.2)
*Loss of S100 n (%)*				
Absent Present	32 (84.2) 25 (75.8)	6 (15.8) 8 (24.2)	0.553	38 (53.5) 33 (46.5)
*Loss of SDHB n (%)*				
Absent Present	48 (85.7) 9 (60)	8 (14.3) 6 (40)	**0.037**	56 (78.9) 15 (21.1)
*MCM6 score n (%)*				
≤ 3 (low expression) > 3 (high expression)	21 (91.3) 36 (75)	2 (8.7) 12 (25)	0.094	23 (32.4) 48 (67.6)

Statistically significant values are in bold

In the multivariate Cox regression analysis, combining all histopathological and immunohistochemical features, SDHB loss, mitoses > 3/10 HPF, and larger tumor size (> 5.1 cm) were found to be associated with lower metastasis-free survival in PCC/PGL patients (Table 4).

**Table 4:** Cox regression analysis of Metastasis-Free survival in PCC/PGLs patients

Multivariate analysis	Hazard ratio (95% Cl)	*P* value
*Loss of SDHB*		
Absent Present	(Reference) 4.50 (1.42–14.32)	**0.011**
*Mıtosıs*		
≤ 2/10 HPF > 3/10 HPF	(Reference) 3.45 (1.09–10.89)	**0.035**
*Tumor sıze*		
≤ 5 cm > 5 cm	(Reference) 1.28 (1.02–1.60)	**0.030**

Statistically significant values are in bold

## Discussion

Today, it is believed that all PCCs and PGLs possess malignant potential. While the typical histopathological features of these tumors are highly distinctive, they exhibit significant variation in histological architecture and cytological appearance, making them intriguing. In addition, their invasive characteristics—such as vascular invasion, capsular invasion, and extension into adipose tissue and adjacent organs—are well recognized. Unfortunately, histopathological appearance can be misleading, as even benign-appearing tumors may metastasize years after the initial surgery. Consequently, various histopathological algorithms and scoring systems have been developed over the years to assess metastasis risk. Among the most commonly used are the PASS [13] and the GAPP system [14] although a universally recommended scoring system by the WHO is not yet available.

In recent years, molecular pathway discoveries have highlighted that the most significant predictor of aggressive clinical behavior is the presence of germline SDHB mutations [19, 20]. In our study, the immunohistochemical loss of SDHB was associated with distant organ metastasis in both univariate and multivariate analyses. Another molecular anomaly linked to aggressive clinical behavior is MAML3 fusion, which has been reported in approximately 5% of PGLs [10]. A large-scale study by Nathaniel et al. reported nuclear overexpression of the MAML3 immunohistochemical marker in PGLs with MAML3 fusion anomalies [12]. Monteagudo M et al. demonstrated that PPGLs with MAML3 fusion exhibit a distinct transcriptional and methylation profile, which is associated with aggressive tumor behavior. Additionally, in these tumors, they identified PD-L1 overexpression, elevated expression of neuroendocrine-to-mesenchymal transition markers, MYC-target genes, and angiogenesis-related genes. MAML3 tumors present a distinctive microenvironment, characterized by a rich vasculature and a unique immune profile, which offers new opportunities for targeted and combination therapies [21]. However, we did not observe MAML3 overexpression in any of the cases in our series. This could be related to the small sample size, and molecular analysis is needed to confirm the absence of fusion to make definitive conclusions.

The MCM2-7 protein complex is found in proliferative cells and is associated with the initiation of DNA replication [22]. As a result, it appears to be an attractive alternative to Ki-67, which is a strong predictor in most cancers. The literature has demonstrated overexpression of MCM2, MCM4, and MCM6 in various cancers [23–27]. However, as far as we are aware, this is the second study to investigate the relationship between PGL and MCM6. In the study conducted by Pierre et al. [15], a higher rate of metastasis was reported in cases with MCM6 expression ≥ 30% (*p* = 0.004). However, this study does not specify the method used for counting MCM6 (such as whether staining intensity was considered, how many cells were counted, or what was accepted as a hotspot or mean). Furthermore, there is no mention of staining intensity. Consequently, an H-score was not provided, and it appears that only the staining percentage was calculated. In our study, MCM6 overexpression was observed in 67.6% of cases. Although distant organ metastasis was more common in cases with overexpression compared to those without, but this difference was not statistically significant. This may be due to the small sample size or short follow-up period.

Despite numerous studies investigating the potential prognostic role of clinical, biochemical, genetic, and histopathological features in PCC/PGL, no single marker has yet been identified that can reliably guide clinical follow-up by predicting tumor recurrence [28, 29]. Consistent with the literature, our study found that PGLs had a significantly higher rate of distant organ metastasis. In the literature, histological features such as tumor necrosis, > 3 mitoses/10 HPF, high cellularity, and capsular, vascular, or adipose tissue invasion have been proposed as predictors of more aggressive tumors [30]. However, while predictive markers like increased mitosis and vascular invasion are relatively rare, capsular or adipose tissue invasion is not always associated with a metastatic nature [31]. The Ki-67 proliferation index, while having high specificity as a marker of poor prognosis, is characterized by low sensitivity, as nearly half of metastatic PGLs exhibit a Ki-67 index of < 2–3% [32]. In our study, no significant relationship was found between the Ki-67 index and distant organ metastasis. Although a higher metastasis rate was observed in cases with a Ki-67 index > 3% (28%), the rate was still substantial in cases with a Ki-67 index < 1% (18.5%). This may be due to the fact that only a limited number of cells are in the Ki-67 expression phase during the cell cycle [32].

Due to the low prevalence of PCC/PGL, it is challenging to accumulate sufficient clinical experience to establish reliable prognostic markers. Although no definitive characteristic has been identified to predict metastasis, large tumor size (> 5 cm), extra-adrenal localization, and SDHB loss are considered high-risk factors for metastasis [33]. In our study, univariate analysis revealed that extra-adrenal localization, vascular invasion, capsular invasion, nuclear pleomorphism, hyperchromasia, and confluent necrosis were associated with distant organ metastasis. Furthermore, multivariate analysis identified large tumor size (> 5.1 cm), presence of > 3 mitoses/10 HPF, and SDHB loss as being associated with lower metastasis-free survival. All these features are long-established and widely accepted prognostic markers in PCC/PGL. Our study is one of the few articles investigating the overexpression of MCM6 in PCC/PGL, a novel molecule that has been studied in various malignancies and shown to have prognostic value in many tumors. Although a higher rate of distant organ metastasis was observed in cases with MCM6 overexpression in our study, this difference was not statistically significant. This may be due to the small sample size and short follow-up period. Larger studies are needed to further investigate the prognostic significance of MCM6 in PCC/PGL.

## Conclusion

The main limitations of our study are the lack of genetic characteristics, the short follow-up duration, and the small sample size. Although several scoring systems with similar characteristics have been proposed for PCC/PGL, the absence of a definitive parameter to accurately determine metastatic potential remains a significant issue in patient follow-up. In our study, while most of the previously described histopathological features were found to be associated with metastasis in univariate analyses, in multivariate analysis, high mitosis, tumor size, and SDHB loss emerged as the key factors. Although MCM6 overexpression is promising, it needs to be supported by studies with larger patient cohorts. The Ki-67 index is quite ineffective in predicting metastasis, while sustentacular cell loss is a feature that occurs more frequently in PGL cases compared to PCC cases. This finding strongly suggests that S100 loss is more related to localization than to metastasis. Furthermore, the recently identified MAML3 fusion is also associated with poor prognosis. In our study, only immunohistochemical analysis was performed, and no expression was detected in any case. However, this result was not confirmed by molecular analysis. In conclusion, while histopathological and immunohistochemical features provide guidance in PCC/PGL cases, they are still insufficient in predicting prognosis. The inclusion of new biomarkers, such as MAML3 and MCM6, which have recently been highlighted for their prognostic value, in routine practice could shed light on what we do not yet know about these tumors. Larger cohort studies are nee

## Data Availability

Not applicable.
